# Lung function impairment and eosinophilia in patients with eosinophilic chronic rhinosinusitis

**DOI:** 10.1016/j.jacig.2025.100550

**Published:** 2025-08-05

**Authors:** Yuki Sonoda, Yoshimasa Imoto, Ayako Maegawa, Anna Shimizu, Masanori Kidoguchi, Rikako Gozawa, Keisuke Koyama, Naoto Adachi, Taiyo Morikawa, Yuto Miyazaki, Takahiro Tokunaga, Masafumi Sakashita, Shigeharu Ueki, Takechiyo Yamada, Shigeharu Fujieda

**Affiliations:** a Department of Otorhinolaryngology, Head & Neck Surgery, Faculty of Medical Sciences, University of Fukui, Fukui, Japan; b Department of Otorhinolaryngology, Shinseikai Toyama Hospital, Toyama, Japan; c Department of General Internal Medicine and Clinical Laboratory Medicine, Akita University Graduate School of Medicine, Akita, Japan; d Department of Otorhinolaryngology, Head & Neck Surgery, Faculty of Medical Sciences, University of Akita, Akita, Japan

**Keywords:** Eosinophilic chronic rhinosinusitis, lung function, eosinophils, type 2 cytokines, asthma, nasal polyps

## Abstract

**Background:**

Eosinophilic chronic rhinosinusitis (ECRS) is characterized by intense eosinophil infiltration in nasal polyps (NPs), related to asthma comorbidity and elevated circulating eosinophil levels. The mechanism by which these systemic components affect type 2 inflammation in NPs is poorly understood.

**Objective:**

We sought to evaluate the relationship between lung function and eosinophilia in chronic rhinosinusitis and assess whether ECRS reflects lower airway involvement and systemic eosinophilic inflammation.

**Methods:**

We examined 198 patients with chronic rhinosinusitis. Lung function was assessed preoperatively using spirometry and fractional exhaled nitric oxide (Feno). Patients were classified into ECRS and non-ECRS groups on the basis of the Japanese Epidemiological Survey of Refractory Eosinophilic Chronic Rhinosinusitis score, and patients with odontogenic maxillary sinusitis (OMS) were included for comparison. mRNA expressions of inflammatory cytokines in NPs were measured by quantitative real-time PCR.

**Results:**

FEV_1_/forced vital capacity ratio, predicted FEV_1_/forced vital capacity, and predicted maximum midexpiratory flow were significantly lower in the ECRS group than in the non-ECRS and OMS groups. Feno levels were significantly higher in the ECRS group. The Japanese Epidemiological Survey of Refractory Eosinophilic Chronic Rhinosinusitis score correlated with lung function and Feno. Notably, some patients with ECRS showed impaired respiratory function and elevated Feno levels without a documented asthma diagnosis, suggesting possible undiagnosed asthma. Gene expression levels of type 2 cytokines in NPs were elevated in patients with ECRS and in those with peripheral eosinophilia greater than 5%.

**Conclusions:**

Respiratory function was significantly lower in patients with ECRS without an asthma diagnosis compared with non-ECRS and OMS groups. Enhanced eosinophilic inflammation in NPs may affect the lower airways, suggesting an eosinophilic united airway disease.

## Graphical abstract

**Figure:**
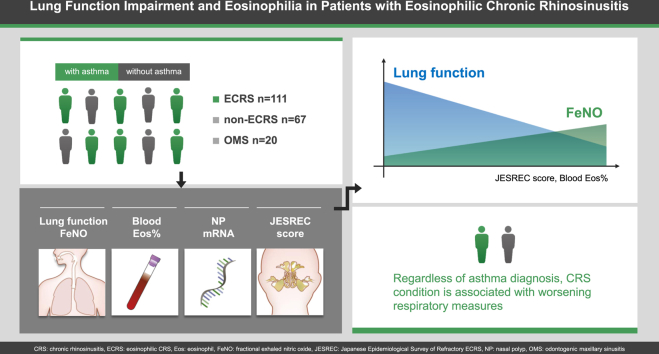

Chronic rhinosinusitis (CRS) is a common chronic upper airway disease that impairs the quality of life of patients by causing headaches, nasal congestion, and hyposmia. Phenotypically, CRS is divided into 2 categories on the basis of the presence or absence of nasal polyps (NPs), namely, CRS with NPs (CRSwNPs) and CRS without NPs. Patients with CRS show high comorbidity of asthma.^1^, ^2^, ^3^ CRS displays a heterogeneous inflammatory spectrum regarded as endotypes and clinical phenotypes.^4^, ^5^, ^6^, ^7^, ^8^ A new classification of CRS on the basis of basic and clinical research has been proposed.^9^

A multicenter retrospective study in Japan, the Japanese Epidemiological Survey of Refractory Eosinophilic Chronic Rhinosinusitis (JESREC) study, examined the clinical and pathological features of patients with CRS who underwent sinus surgery.^10^ According to this study, eosinophilic CRS (ECRS) can be diagnosed on the basis of the total score, which includes bilateral disease, blood eosinophils (>5%), ethmoid disease, the existence of NPs, and tissue eosinophils. The JESREC study also revealed that elevated blood eosinophil count, asthma, and nonsteroidal anti-inflammatory drug–exacerbated respiratory disease (N-ERD) were all associated with disease recurrence and the need for further surgical intervention. In addition, tissue eosinophilia is a strong predictor of CRS severity,^11^, ^12^, ^13^ and the cooccurrence of asthma and CRS has implications for the severity of both conditions.^14^^,^^15^ Asthma is the most common, chronic inflammatory airway disease,^16^ and patients with CRS show a higher risk of asthma,^17^^,^^18^ which impairs their quality of life. Population and/or patient-reported data consistently show that asthma is underdiagnosed and overdiagnosed because the diagnosis is usually based on symptoms alone without respiratory assessments through spirometry.^19^ ECRS is considered a severe form of CRS because of high recurrence accompanied by enhanced eosinophilic inflammation. However, certain clinical questions remain unanswered, such as whether there are latent pathological lesions in the lower respiratory tract of patients with ECRS and the relationship between the JESREC score or peripheral eosinophils and lower respiratory tract inflammation in patients with CRS, which the present study aimed to address.

Previous studies have highlighted that endoscopic sinus surgery (ESS) results in reductions in incident asthma and improvements in asthma-associated quality of life.^20^, ^21^, ^22^ The results also indicate that CRS treatment may influence asthma occurrence and even prevent its onset, suggesting the significance of shared inflammation and contiguous airways.^23^ We aimed to investigate preoperative lung function in patients with CRS to understand better the clinical questions mentioned earlier. We also demonstrated the relationship between upper and lower respiratory tract inflammation on the basis of the JESREC criteria by focusing on eosinophilia.

## Methods

### Patient recruitment and clinical sample collection

Sinus disease was diagnosed on the basis of patient history, clinical examination, nasal endoscopy, and computed tomography (CT) of the sinuses according to the guidelines of the European Position Paper on Rhinosinusitis and Nasal Polyps.^9^ Patients with CRS were recruited before surgery from the University of Fukui Department of Otorhinolaryngology, Head & Neck Surgery, Fukui, Japan, and all patients provided written informed consent before sample collection. Patients with established immunodeficiency, pregnancy, coagulation disorders, classic allergic fungal sinusitis, fungal sinusitis, eosinophilic granulomatosis with polyangiitis, or cystic fibrosis were excluded from the study. All patients scheduled for surgery previously failed to respond to adequate trials of conservative medical therapy (prolonged antibiotic regimens, nasal steroid sprays, oral steroids, saline irrigation, and decongestants) for symptom control. This study was performed in compliance with the Declaration of Helsinki and Good Clinical Practice guidelines, with previous approval from the University of Fukui Ethics Committee (20180023).

The JESREC scoring criteria for diagnosing ECRS represent a scoring system that assesses unilateral or bilateral disease, the presence of NPs, ethmoid-dominant CT shadows, and eosinophil ratio in the peripheral blood, as described previously.^10^ ECRS was defined as a total score of 11 or higher. Patients with CRS due to odontogenic maxillary sinusitis (OMS), a subtype of unilateral maxillary sinusitis caused by inflammation from neighboring maxillary teeth or as a result of iatrogenic damage during dental interventions,^24^ were included in the comparison group. These diseases were diagnosed preoperatively. In this study, treatment of ECRS using steroids was discontinued at least 1 month before surgery. Therefore, the use of oral steroids in these patients was intended for the treatment of only asthma.

Spirometry tests were performed on all patients as part of preoperative examinations 2 weeks before surgery using CHESTAC-8900 (Chest, Tokyo, Japan). There were no restrictions on medications other than steroids for the preoperative examination. In this study, we used FEV_1_/forced vital capacity (FVC) as the percentage of FEV_1_ in FVC, and maximum midexpiratory flow (MMF; forced expiratory flow at 25%-75% of FVC) as the average expiratory flow during the middle 50% of the FVC. Predicted values for FEV_1_/FVC and MMF were calculated using the Global Lung Function Initiative reference equations, adjusted for age, sex, height, and ethnicity.^25^ The percentage predicted values were obtained by dividing the measured values by the predicted values and multiplying by 100. Fractional exhaled nitric oxide (Feno) levels were measured on the basis of the American Thoracic Society/European Respiratory Society guidelines using Sievers Nitric Oxide Analyzer (NOA 280i; GE Analytical Instruments, Beverly, Mass).^26^ Measurements were performed at least thrice, and the mean of the 3 values was calculated for the analysis.

Patients were considered to have asthma if they had an asthma diagnosis and had symptoms documented by an allergist, pulmonologist, or otolaryngologist.

### Study design

All NP specimens and relevant clinical information were collected prospectively from patients undergoing sinus surgery at the University of Fukui. These specimens were stored and managed in accordance with ethical guidelines and institutional protocols. For the present study, specific samples and corresponding clinical data were selected retrospectively, and so the study design and analysis presented here are retrospective in nature.

### Quantitative real-time PCR for nasal tissue

NP tissues from patients with CRS were obtained during surgery. Details of the patient characteristics are provided in Table E1 (in the Online Repository available at www.jaci-global.org). The tissues were immediately placed in a stabilization reagent (RNAlater; Invitrogen, Valencia, Calif), and total RNA was extracted using NucleoSpin RNA II (Macherey-Nagel, Bethlehem, Pa) with DNase I (Invitrogen, Carlsbad, Calif), according to the manufacturers’ instructions. The quality of total RNA obtained from the sinus tissue was assessed with a 2100 Bioanalyzer (Agilent Technologies, Santa Clara, Calif) using an RNA 6000 Nano LabChip (Agilent Technologies). The RNA integrity number was used to assess the overall quality, and samples with RNA integrity values greater than 7.0 were considered suitable for downstream applications.

Single-strand cDNA was synthesized using a high-capacity cDNA Reverse Transcription Kit (Thermo Fisher Scientific, Waltham, Mass). Semiquantitative real-time PCR (RT-PCR) was performed with the TaqMan method using an Applied Biosystems StepOnePlus RT-PCR system (Thermo Fisher Scientific) in 15-μL reactions (7.5 μL of 2× TaqMan Master mix [Thermo Fisher Scientific] and 0.75 μL of 20× primer and probe mixture). Probes for *IL-4*, *IL-5*, *IL-13*, *arachidonate 15-lipoxygenase* (*ALOX15*), *cystatin SN* (*CST1*), *periostin* (*POSTN*), *C-C motif chemokine ligand 26* (*CCL26*), *IFN-γ*, and *glyceraldehyde 3-phosphate dehydrogenase* (*GAPDH*) were purchased from Thermo Fisher Scientific. Quantified aliquots of purified PCR fragments of the target genes were serially diluted and used as standards in each experiment to determine the exact copy numbers of the target genes. Aliquots of cDNA, equivalent to 10 ng of total RNA, were used for RT-PCR. Gene expression profiles in NPs were analyzed using the ΔΔCT method for relative quantification. The mRNA expression levels were normalized to the median expression level of the housekeeping gene *GAPDH*.

### Statistical analysis

Differences between groups were analyzed using the Kruskal-Wallis test, the Dunnett *post hoc* test, and the Mann-Whitney *U* test. Correlations were assessed using the Spearman rank correlation. Statistical significance was set at a *P* value less than .05. All statistical analyses were performed using GraphPad Prism 10.0 (GraphPad Software, La Jolla, Calif).

## Results

### Characteristics of the patients

We enrolled 198 patients in this study. Table I provides the characteristics of all the study patients. No significant differences were observed between the patients in the ECRS and OMS groups in terms of sex, age, smoking rate, or body mass index (BMI). In the non-ECRS group, the age was slightly higher than that in the ECRS group. However, sex, smoking rate, or BMI did not differ significantly. Total serum IgE levels in the ECRS group were significantly higher than those in the non-ECRS group (*P* < .01). Of the patients in the ECRS group, 47.8% had asthma as a comorbidity, and this proportion was significantly higher than that in the non-ECRS (*P* < .0001) and OMS (*P* < .0001) groups. The N-ERD rate was also significantly higher in the ECRS group than in the non-ECRS group (*P* < .01), with all patients having a concurrent diagnosis of asthma. In addition, patients with ECRS showed higher use of inhaled corticosteroids (vs non-ECRS, *P* < .0001; vs OMS, *P* < .0001) and intake of oral steroids (vs non-ECRS, *P* < .01; vs OMS, *P* < .01). Oral steroids were used for the treatment of both ECRS and asthma, but patients with ECRS were instructed to discontinue oral steroid treatment at least 1 month before surgery.

**Table I:** Characteristics of the patients

Characteristics	OMS (n = 20)	Non-ECRS (n = 67)	ECRS (n = 111)
Sex: male/female	12/8	41/26	83/28
Age (y), mean ± SD	54.5 ± 15.7	58.3 ± 16.0	54.4 ± 13.1∗
Smoking, yes/no	9/11	38/29	75/36
BMI (kg/m^2^), mean ± SD	23.9 ± 4.8	23.4 ± 3.3	23.8 ± 3.0
Asthma, yes/no	0/20	2/65	53/58†,‡
N-ERD, yes/no	0/20	0/67	14/97§
Inhaled corticosteroids, yes/no	0/20	2/65	47/64†,‡
Oral steroids, yes/no	0/20	5/62	28/83§,‖
NPs, yes/no	5/15	29/38	111/0†,‡
Eosinophils in peripheral blood (%), mean ± SD	2.3 ± 1.7	3.2 ± 2.2	8.0 ± 5.1†,‡
Total IgE, mean ± SD	106.0 ± 408.7	296.1 ± 578.0	483.7 ± 841.8§
JESREC score, mean ± SD	0.0 ± 3.6	5.3 ± 3.3¶	14.4 ± 2.2†,‡

∗ *P* < .05 vs non-ECRS.

† *P* < .0001 vs OMS.

‡ *P* < .0001 vs non-ECRS.

§ *P* < .01 vs non-ECRS.

‖ *P* < .01 vs OMS.

¶ *P* < .05 vs OMS.

### Lung function characteristics

**Fig 1:**
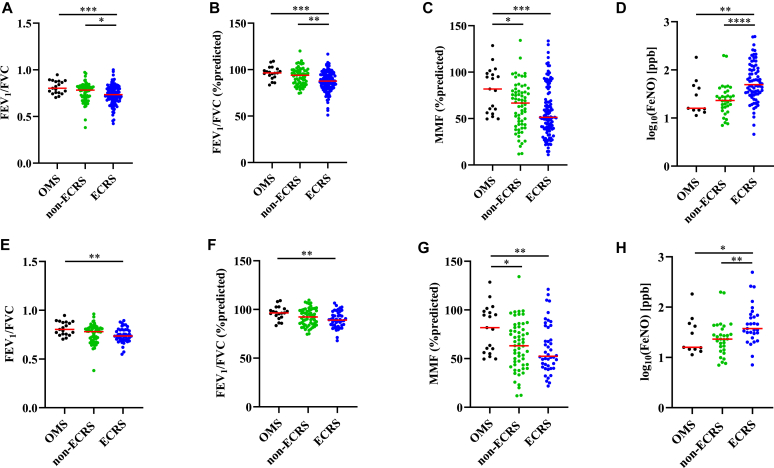
**A-H,** Lung function and Feno levels before surgery. Dot plots illustrate individual data points: *blue*, ECRS; *green*, non-ECRS; and *black*, OMS. FEV_1_/FVC (Fig 1, *A*), predicted FEV_1_/FVC (Fig 1, *B*), predicted MMF (Fig 1, *C*), and Feno (Fig 1, *D*) in all patients (n = 20-111) and in patients without a history of asthma or steroid use (Fig 1, *E-H*) (n = 20-59). ∗*P* < .05; ∗∗*P* < .01; ∗∗∗*P* < .001; ∗∗∗∗*P* < .0001.

Fig 1 shows the lung function results. Compared with the other 2 groups, patients in the ECRS group showed significantly decreased levels of FEV_1_/FVC (ECRS, 0.734 ± 0.102; vs non-ECRS, 0.782 ± 0.134, *P* < .05; vs OMS, 0.803 ± 0.07, *P* < .001; Fig 1, *A*), predicted FEV_1_/FVC (ECRS, 87.7% ± 11.2%; vs non-ECRS, 93.1% ± 11.7%, *P* < .01; vs OMS, 96.4% ± 6.9%, *P* < .001; Fig 1, *B*), and predicted MMF (ECRS, 51.3% ± 27.8%, *P* < .05; vs non-ECRS, 66.7% ± 25.4%, *P* < .05; vs OMS, 81.8% ± 24.3%, *P* < .001; Fig 1, *C*). In addition, patients in the ECRS group had significantly elevated levels of Feno compared with the other 2 groups (ECRS, 42.0 ± 32.0 ppb; vs non-ECRS, 22.9 ± 11.7 ppb, *P* < .0001; vs OMS, 15.8 ± 17.3 ppb, *P* < .01; Fig 1, *D*). Clinically, an FEV_1_/FVC ratio less than 0.7 indicates airflow limitation.^16^ Therefore, we examined the proportion of patients with an FEV_1_/FVC ratio less than 0.7 among all groups. Thirty-seven patients (33.3%) in the ECRS group and 18 patients (26.9%) in the non-ECRS group showed an FEV_1_/FVC ratio less than 0.7; this was significantly higher than that of the patients in the OMS group (vs ECRS, *P* < .05; vs non-ECRS, *P* < .05; Fig 1, *A*).

Because oral and inhaled steroids suppress airway type 2 inflammation, they may also affect laboratory values. We selected patients who had not received inhaled or oral steroids and those who had not been previously diagnosed with asthma or N-ERD to evaluate lung function accurately. We defined these patients as “patients without a history of asthma nor steroid use,” and their details are provided in Table II. The results showed that FEV_1_/FVC and predicted FEV_1_/FVC levels in patients with ECRS (FEV_1_/FVC, 0.736 ± 0.075; predicted FEV_1_/FVC, 89.1% ± 8.5%) were significantly decreased compared with those in the OMS group (FEV_1_/FVC, 0.803 ± 0.07, *P* < .01; predicted FEV_1_/FVC, 96.4% ± 6.9%, *P* < .01), but not those in the non-ECRS group (FEV_1_/FVC, 0.782 ± 0.082; predicted FEV_1_/FVC, 92.4% ± 8.8%; Fig 1, *E* and *F*). Eleven patients (25.0%) in the ECRS group and 14 patients (23.7%) in the non-ECRS group showed an FEV_1_/FVC ratio less than 0.7, and this proportion was significantly higher than that in the OMS group, in which no patient showed an FEV_1_/FVC ratio less than 0.7 (vs ECRS, *P* < .05; vs non-ECRS, *P* < .05; Fig 1, *E*). In contrast, the values of predicted MMF in the ECRS and non-ECRS groups were significantly decreased compared with those in the OMS group (ECRS, 52.4% ± 25.7%; non-ECRS, 63.2% ± 25.1%; vs OMS, 81.8% ± 24.3%, *P* < .01; Fig 1, *G*). Furthermore, patients with ECRS had elevated Feno levels compared with those in the non-ECRS and OMS groups (ECRS, 35.2 ± 28.3 ppb; vs non-ECRS, 22.9 ± 12.3 ppb, *P* < .01; vs OMS, 15.8 ± 17.3 ppb, *P* < .05; Fig 1, *H*).

**Table II:** Characteristics of the patients without a history of asthma and steroid use

Characteristics	OMS (n = 20)	Non-ECRS (n = 59)	ECRS (n = 44)
Sex: male/female	12/8	37/22	10/34
Age (y), mean ± SD	54.5 ± 15.7	57.8 ± 16.4	56.7 ± 11.8
Smoking, yes/no	9/11	34/25	31/13
BMI (kg/m^2^), mean ± SD	23.9 ± 4.8	23.5 ± 3.1	24.4 ± 2.9
NPs, yes/no	5/15	27/32	44/0∗,†
Eosinophils in peripheral blood (%), mean ± SD	2.3 ± 1.7	3.4 ± 2.3	6.4 ± 2.3∗,†
Total IgE, mean ± SD	106.0 ± 408.7	68.3 ± 609.4	206.5 ± 284.8
JESREC score, mean ± SD	0.0 ± 3.6	5.3 ± 3.2‡	13.5 ± 2.0∗,†

∗ *P* < .0001 vs OMS.

† *P* < .0001 vs non-ECRS.

‡ *P* < .05 vs OMS.

**Fig 2:**
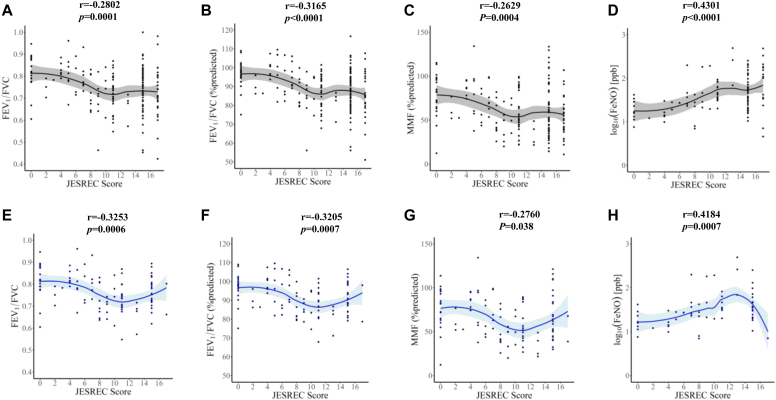
**A-H,** Correlation of JESREC score with FEV_1_/FVC (Fig 2, *A*), predicted FEV_1_/FVC (Fig 2, *B*), predicted MMF (Fig 2, *C*), and Feno (Fig 2, *D*) before surgery in all patients (n = 198) and in patients without a history of asthma or steroid use (Fig 2, *E-H*) (n = 123). The correlation was assessed using the Spearman rank correlation test.

**Fig 3:**
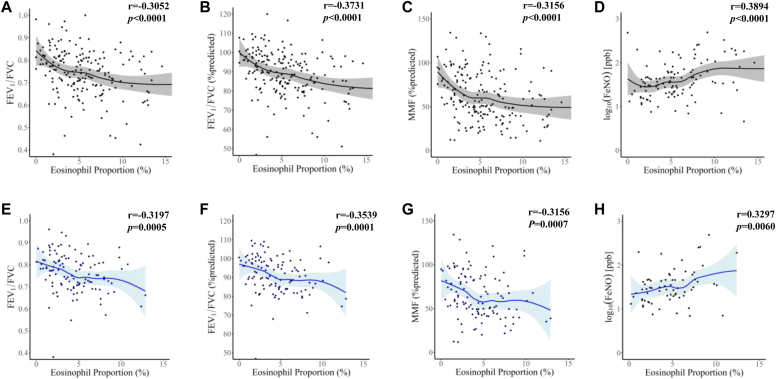
**A-H,** The correlation of peripheral blood eosinophil proportion with FEV_1_/FVC (Fig 3, *A*), predicted FEV_1_/FVC (Fig 3, *B*), predicted MMF (Fig 3, *C*), and Feno (Fig 3, *D*) before surgery in all patients (n = 198) and in patients without a history of asthma or steroid use (Fig 3, *E-H*) (n = 123). The correlation was assessed using the Spearman rank correlation test.

Next, we demonstrated the correlation between the JESREC score and lung function. In all patients, the JESREC score was significantly correlated with FEV_1_/FVC (*r* = −0.2802; *P* = .0001), predicted FEV_1_/FVC (*r* = −0.3165; *P* < .0001), predicted MMF (*r* = −0.2629; *P* = .0004), and Feno (*r* = 0.4301; *P* < .0001) (Fig 2, *A*-*D*). The results were similar, even in patients with untreated, undiagnosed asthma (FEV_1_/FVC, *r* = −0.3253, *P* = .0006; predicted FEV_1_/FVC, *r* = −0.3205, *P* = .0007; predicted MMF, *r* = −0.2760, *P* < .05; Feno, *r* = 0.4184, *P* = .0007) (Fig 2, *E*-*H*). Similar to the JESREC score, the peripheral blood eosinophil count correlated with lung function in all cases (Fig 3, *A*-*D*) and in patients without a history of asthma or steroid use (Fig 3, *E*-*H*).

### Gene expression analysis of NPs

**Fig 4:**
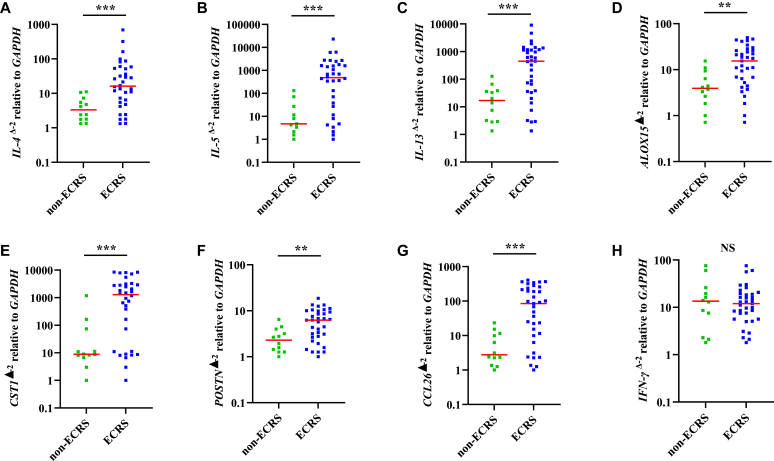
**A-H,** Gene expression levels of type 2 cytokines in NP tissue. The expression levels of *IL-4* (Fig 4, *A*), *IL-5* (Fig 4, *B*), *IL-13* (Fig 4, *C*), *ALOX15* (Fig 4, *D*), *CST1* (Fig 4, *E*), *POSTN* (Fig 4, *F*), and *CCL26* (Fig 4, *G*) were significantly elevated in patients with ECRS, but not of *IFN-γ* (Fig 4, *H*). *NS*, Not significant. ∗∗*P* < .01; ∗∗∗*P* < .001.

**Fig 5:**
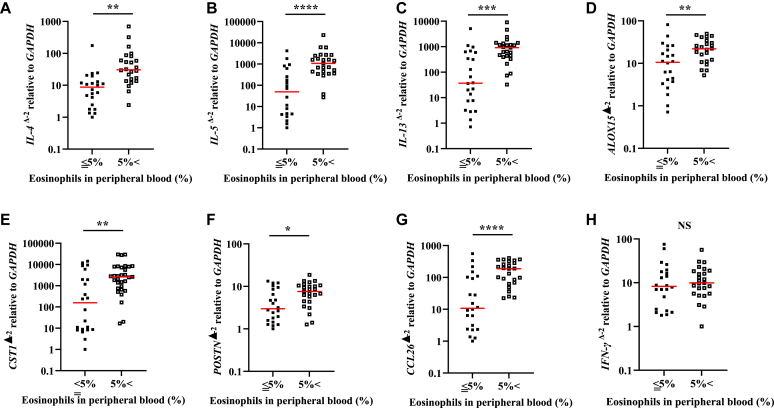
**A-H,** Gene expression levels of type 2 cytokines in NP tissue. The expression levels of *IL-4* (Fig 5, *A*), *IL-5* (Fig 5, *B*), *IL-13* (Fig 5, *C*), *ALOX15* (Fig 5, *D*), *CST1* (Fig 5, *E*), *POSTN* (Fig 5, *F*), and *CCL26* (Fig 5, *G*) were significantly elevated in patients with peripheral eosinophilia greater than 5%, but not of *IFN-γ* (Fig 5, *H*). *NS*, Not significant. ∗*P* < .05; ∗∗*P* < .01; ∗∗∗*P* < .001; ∗∗∗∗*P* < .0001.

Gene expression analysis of NPs showed that type 2–related cytokines such as *IL-4*, *IL-5*, *IL-13*, *ALOX15*, *CST1*, *POSTN*, and *CCL26* were significantly elevated in patients with ECRS (Fig 4). According to the JESREC study, an eosinophil count greater than or equal to 5% in peripheral blood has a significant value in defining ECRS,^10^ and eosinophilia is also a clinical feature of eosinophilic airway diseases.^27^ We studied the gene expression patterns of NPs to understand better the significance of peripheral eosinophils in the NP endotypes. We demonstrated that gene expression patterns depend on the peripheral eosinophils. The results revealed that unlike *IFN-γ*, *IL-4*, *IL-5*, *IL-13*, *ALOX15*, *CST1*, *POSTN*, and *CCL26* were significantly elevated in patients with eosinophilia greater than 5% (Fig 5). We also demonstrated correlations between gene expression levels in NPs and the proportion of eosinophils in peripheral blood. *IL-4*, *IL-5*, *IL-13*, *ALOX15*, *CST1*, *POSTN*, and *CCL26* significantly correlated with eosinophils in peripheral blood (Table III).

**Table III:** Correlation with eosinophils in peripheral blood

Gene	*r*	*P* value
*IL-4*	0.6941	<.0001
*IL-5*	0.5664	<.0001
*IL-13*	0.5884	<.0001
*ALOX15*	0.3916	<.01
*CST1*	0.3801	<.01
*POSTN*	0.4276	<.01
*CCL26*	0.5886	<.0001
*IFN-γ*	0.0504	NS

*NS*, Not significant.

## Discussion

In the present study, patients with ECRS showed significantly decreased FEV_1_/FVC, predicted FEV_1_/FVC, and predicted MMF levels compared with those in the non-ECRS and OMS groups (Fig 1). This study also revealed that patients with ECRS showed a higher prevalence of asthma (47.8%), and 33.3% of patients showed FEV_1_/FVC less than 0.7 despite taking oral and/or inhaled corticosteroids (Fig 1, *A*). Furthermore, we observed decreased levels of FEV_1_/FVC, predicted FEV_1_/FVC, and predicted MMF and elevated Feno levels in patients with ECRS who had not received inhaled or oral steroids nor had been previously diagnosed with asthma, and this may indicate aspects of latent lower respiratory tract inflammation (Fig 1, *E*-*H*). Because significant correlations were observed between the JESREC score and lung function data (Fig 2), ECRS diagnosis based on the JESREC score may potentially reflect systemic eosinophilic airway inflammation.

This study showed frequent comorbidity of ECRS and asthma. However, the comorbidity rate varied depending on the reports, and this may be due to different components such as phenotype, severity, and study population of CRS.^28^ In the present study, the proportion of patients with ECRS who used steroids was also higher (inhaled, 42.3%; oral, 25.2%) than of those who used steroids in the non-ECRS and OMS groups. Because patients with severe asthma require steroids,^29^ the patients surveyed in this study who were using steroids may be considered to have severe asthma. Asthma-onset pattern may influence severity, because patients who required steroids were more likely to have late-onset asthma^18^; however, we did not examine asthma onset in this study, and this may be one of the limitations of our study. Notably, most previous reports did not accurately diagnose asthma or assess respiratory function because the clinical information was either from the patient or a mixture of patients who were receiving steroids and those who were not. Therefore, we analyzed lung function in patients who had not received inhaled or oral steroids or who had been previously diagnosed with asthma. Although the differences between ECRS and non-ECRS groups were not statistically significant, the stepwise decline in MMF may highlight its potential sensitivity to airway changes between ECRS and non-ECRS groups. FEV_1_/FVC is a general indicator of overall airflow limitation, whereas MMF reflects midexpiratory flow and may be more responsive to change in medium- to small-sized airway caliber or inflammation. This can also be explained by the heterogeneity of CRSwNP, with non-ECRS potentially reflecting a gradient that leans toward ECRS.^7^^,^^27^ Decreased levels of predicted MMF in patients with ECRS are also supported by the study by Ragab et al,^30^ which showed a significant proportion of patients with CRSwNP having some form of lower airway involvement.^30^ Further clinical and pathological studies are required to understand the precise mechanisms better.

Feno levels reflect type 2 airway inflammation, which is associated with the extent of eosinophilic inflammation in the tissue.^31^ Feno displays high potential in diagnosing asthma and is a biomarker of type 2 airway inflammation and lung function.^32^^,^^33^ Emerging reports have highlighted that Feno reflects disease severity and the degree of inflammation in eosinophilic airway disease.^34^, ^35^, ^36^ The present study revealed that the Feno levels in the ECRS group were significantly higher than those in the non-ECRS and OMS groups (Fig 1, *D* and *H*). Because Feno levels are affected by sex, atopy, and smoking,^33^ we demonstrated the differences in these factors between the ECRS and non-ECRS groups. Regardless of a previous diagnosis of asthma or steroid use, no significant differences were found in sex or smoking status (Table II). These results may indicate that patients with ECRS have enhanced eosinophilic inflammation in the lower airway, accompanied by elevated Feno levels. The best cutoff value of Feno for diagnosing eosinophilic airway disease in different categories of patients has been discussed,^16^^,^^37^, ^38^, ^39^ and the results indicate that there may be significant proportions of patients with ECRS who have eosinophilic inflammation in the lower respiratory tract and the nasal and paranasal sinuses.

Elevated peripheral blood eosinophil levels are also regarded as clinical markers of type 2 inflammation, which has a large impact on ECRS diagnosis.^10^ Blood eosinophil count cannot define asthma because it lacks sensitivity in diagnosing asthma; however, it can provide information regarding the phenotype or severity of asthma.^16^^,^^40^, ^41^, ^42^ This study also demonstrated a correlation between peripheral blood eosinophil count and lung function, suggesting that the blood eosinophil count reflects the extent of lower airway inflammation (Fig 3). Reportedly, higher blood eosinophil counts are associated with male sex, obesity, and smoking in healthy individuals.^43^ We investigated these factors in patients with ECRS by comparing them with those in the non-ECRS groups and found no significant differences in sex, BMI, or smoking between patients in the ECRS and non-ECRS groups (Tables I and II). Peripheral blood eosinophilia greater than 300/μL is common in patients with asthma and is associated with poor asthma control and frequent exacerbations of adult-onset severe asthma.^27^ Because JESREC criteria define eosinophilia by the proportion of eosinophils in peripheral blood, a value of 5% or higher is a significant value that can define ECRS.^10^ We studied how the proportion of eosinophils in peripheral blood reflects the number by calculating the number of whole white blood cells. An eosinophil count of 5% in peripheral blood reflects approximately 300/μL of eosinophils (see Fig E1 in this article’s Online Repository at www.jaci-global.org). Notably, enhanced peripheral eosinophilia is associated with type 2 cytokine gene expression in the sputum of patients with asthma.^44^ Differences in gene expression patterns in NPs between patients with and without ECRS have been reported^6^^,^^45^; however, the significance of peripheral blood eosinophils for gene expression in NPs has not been well studied. *IL-4*, *IL-5*, *IL-13*, *ALOX15*, *CST1*, *POSTN*, and *CCL26* were significantly elevated in patients who had eosinophilia higher than 5%, but not *IFN-γ*. In addition, *ALOX15*, *CST1*, and *POSTN*, which encode 15-lipoxygenase 1, cystatin SN, and periostin, respectively, are related to eosinophilic inflammation, because they contribute to eosinophil accumulation in NP tissues.^45^, ^46^, ^47^, ^48^, ^49^ *CCL26*, also known as eosinophil chemotactic protein or eotaxin-3, recruits eosinophils into inflammatory tissues, thereby contributing to eosinophilia.^50^, ^51^, ^52^ These genes are affected by type 2 cytokines. Hence, enhanced type 2 inflammation results in eosinophilia in the tissues.^53^ Therefore, as a part of the JESREC criteria, an eosinophil count greater than 5% in peripheral blood is significant because it reflects elevated type 2 inflammation in the tissue, similar to asthma. In patients with asthma, peripheral blood eosinophilia is recognized as the most important biomarker for predicting the efficacy of biologics targeting *IL-5*.^54^, ^55^, ^56^ An eosinophil count greater than 5% may be valuable to identify candidates who would benefit most from biological therapy in treating ECRS; however, further studies are required.

Our study had some limitations. First, respiratory function, asthma diagnosis, and peripheral blood eosinophils were investigated preoperatively but not postoperatively. In addition, we did not study the recurrence of CRS, nasal symptoms, changes in respiratory function, or the development of asthma postsurgery. Whether ESS improves asthma and CRS symptoms has been well discussed,^57^ but evidence to indicate that ESS improves pulmonary function in individuals with CRS and asthma was insufficient.^58^ Early diagnosis of sinusitis is associated with new-onset asthma.^59^ Hence, ECRS diagnosis based on the JESREC criteria may be significant from the perspective of early intervention against asthma. Although patients with asthma in our study were diagnosed by a specialist, standardized diagnostic procedures such as bronchodilator reversibility testing or bronchial provocation testing were not systematically performed in all patients. Therefore, the possibility of undiagnosed asthma cannot be completely ruled out in our study. Moreover, it became evident that some patients with decreased respiratory function did not undergo further testing or follow-up after their initial preoperative assessments. This may be because of the absence of respiratory symptoms at the time of evaluation. However, given the risk of future asthma attacks in such individuals, early intervention by respiratory specialists may be recommended. Second, the number of patients with N-ERD was limited (n = 14). Because N-ERD is severe in patients with CRSwNP,^60^ such phenotypes should be closely monitored in the future. Third, we measured Feno levels, but we lacked pathological data from the lungs, such as sputum or bronchoalveolar lavage fluid, which may have strengthened the connection between upper and lower respiratory tract inflammation. Further clinical studies mentioning both phenotypes and endotypes are required to clarify this aspect.

This study revealed that many patients with ECRS, even those without a history of asthma, showed signs of lower respiratory tract involvement, such as decreased lung function and elevated Feno levels, suggesting the possibility of subclinical or undiagnosed asthma. ECRS diagnosis based on the JESREC score may be significant, because it may reflect lower respiratory tract eosinophilic inflammation. Our results also revealed that blood eosinophilia greater than 5% has significant value in ECRS, because type 2 cytokines in NPs were elevated, and the correlation between clinical phenotypes and molecular endotypes may support effective biological therapies. Because various biologics are currently used for treating CRSwNP, early intervention in united eosinophilic airway disease may be more appropriate if the same analysis is performed before and after using these agents.

**Clinical implications**

This study revealed that patients with ECRS diagnosed using the JESREC criteria had latent lower respiratory tract inflammation. Peripheral eosinophilia results in enhanced type 2 inflammation in NPs and impaired lung function in ECRS.

## Disclosure statement

This work was supported by Japan Society for the Promotion of Science 10.13039/501100001691KAKENHI (grant nos. JP17K11355 and 21K09558 to Y.I.), 10.13039/501100013236MSD Life Science Foundation, Public Interest Incorporated Foundation, and 10.13039/100007428Naito Foundation (to Y.I.).

Disclosure of potential conflict of interest: Y. Imoto reports personal fees from 10.13039/100004330GlaxoSmithKline (GSK) and Sanofi as speakers bureau. S. Ueki received honoraria from AstraZeneca, GSK, and Sanofi; and received grants from AstraZeneca, VIB, Cytrill, and Maruho Co, Ltd. T. Yamada received honoraria from Mitsubishi Tanabe Pharma, Kyorin Pharma, Sanofi, and GSK; and received grant support from Sanofi. S. Fujieda reports personal fees from Kyorin, 10.13039/501100012351Mitsubishi Tanabe Pharma, Taiho Pharma, and Sanofi as speakers bureau; and has also served on the advisory board for AstraZeneca, GSK, and Sanofi. The rest of the authors declare that they have no relevant conflicts of interest.

## Supplementary data

Supplementary Fig E1

Supplementary Table E1
