# Supplementary figures and images for: Lung function impairment and eosinophilia in patients with eosinophilic chronic rhinosinusitis
Source: J Allergy Clin Immunol Glob. 2025 Aug 5;4(4):100550. doi: 10.1016/j.jacig.2025.100550 (PMC12446768; doi:10.1016/j.jacig.2025.100550)

Supplementary Figure 1

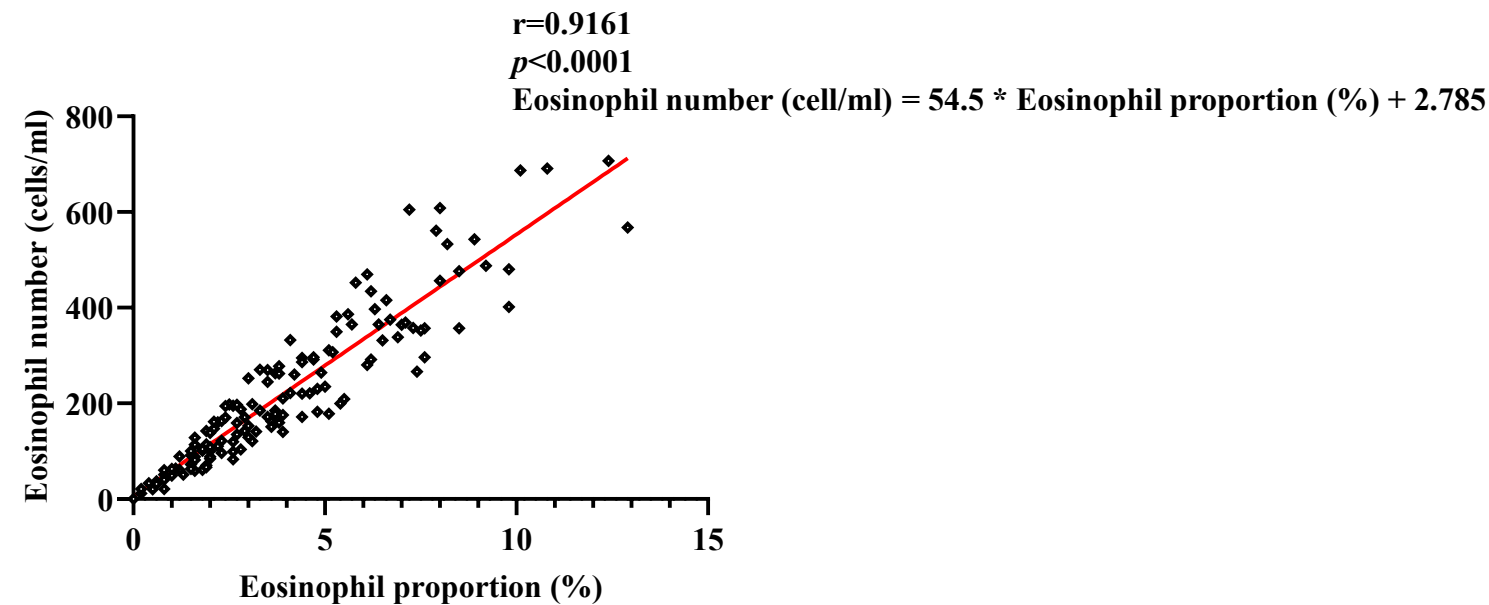

Supplement: Supplementary Fig E1 [file mmc1.pdf]
