# Supplementary material for: Lung function impairment and eosinophilia in patients with eosinophilic chronic rhinosinusitis
Source: J Allergy Clin Immunol Glob. 2025 Aug 5;4(4):100550. doi: 10.1016/j.jacig.2025.100550 (PMC12446768; doi:10.1016/j.jacig.2025.100550)
Supplement: Supplementary Table E1 [file mmc2.docx]

| **Supplementary table 1 Characteristics of the patients** | | |
| --- | --- | --- |
|  | **Non-ECRS** | **ECRS** |
|  | **(n=12)** | **(n=35)** |
| **Sex, male/female** | 9/3 | 29/6 |
| **Age, years** | 52.5+14.6 | 58.0+13.2 |
| **Smoking, yes/no** | 9/3 | 24/11 |
| **BMI** | 23.0+2.6 | 23.0+2.9 |
| **Eosinophils in peripheral blood (%)** | 2.4+1.6 | 6.9+4.0^####^ |
| **Total IgE** | 86.6+653.5 | 232.0+476.8 |
| **JESREC score** | 7.0+2.2 | 15.0+2.4^####^ |

BMI, Body mass index; IgE, Immunoglobulin E; JESREC, Japanese Epidemiological Survey of Refractory Eosinophilic Chronic Rhinosinusitis; ECRS, Eosinophilic chronic rhinosinusitis ####*p*<.0001 vs non-ECRS
