# Supplementary material for: Lung function impairment and eosinophilia in patients with eosinophilic chronic rhinosinusitis
Source: J Allergy Clin Immunol Glob. 2025 Aug 5;4(4):100550. doi: 10.1016/j.jacig.2025.100550 (PMC12446768; doi:10.1016/j.jacig.2025.100550)
Supplement: Supplementary Data [file mmc3.docx]

**Supplementary Figure 1.** Correlation between peripheral blood eosinophil percentage and peripheral blood eosinophil number (eosinophil number [cell/mL] = 54.5 * eosinophil proportion [%] + 2.785).
